# Bcl-2 associated athanogene 5 (Bag5) is overexpressed in prostate cancer and inhibits ER-stress induced apoptosis

**DOI:** 10.1186/1471-2407-13-96

**Published:** 2013-03-01

**Authors:** Anja Bruchmann, Corinna Roller, Tamara Vanessa Walther, Georg Schäfer, Sara Lehmusvaara, Tapio Visakorpi, Helmut Klocker, Andrew C B Cato, Danilo Maddalo

**Affiliations:** 1 Karlsruhe Institute of Technology, Institute of Toxicology and Genetics, Hermann-von-Helmholtz Platz 1, Eggenstein-Leopoldshafen 76344, Germany; 2 Division of Experimental Urology, Department of Urology, Innsbruck Medical University, Innsbruck, Austria; 3 Institute of Biomedical Technology and BioMediTech, University of Tampere and Tampere University Hospital, Tampere FI-33014, Finland

**Keywords:** Unfolded protein response, Cell stress, Endoplasmic reticulum, Apoptosis, Molecular chaperones, Refolding

## Abstract

**Background:**

The Bag (Bcl-2 associated athanogene) family of proteins consists of 6 members sharing a common, single-copied Bag domain through which they interact with the molecular chaperone Hsp70. Bag5 represents an exception in the Bag family since it consists of 5 Bag domains covering the whole protein. Bag proteins like Bag1 and Bag3 have been implicated in tumor growth and survival but it is not known whether Bag5 also exhibits this function.

**Methods:**

Bag5 mRNA and protein expression levels were investigated in prostate cancer patient samples using real-time PCR and immunoblot analyses. In addition immunohistological studies were carried out to determine the expression of Bag5 in tissue arrays. Analysis of Bag5 gene expression was carried out using one-way ANOVA and Bonferroni’s Multiple Comparison test. The mean values of the Bag5 stained cells in the tissue array was analyzed by Mann-Whitney test. Functional studies of the role of Bag5 in prostate cancer cell lines was performed using overexpression and RNA interference analyses.

**Results:**

Our results show that Bag5 is overexpressed in malignant prostate tissue compared to benign samples. In addition we could show that Bag5 levels are increased following endoplasmic reticulum (ER)-stress induction, and Bag5 relocates from the cytoplasm to the ER during this process. We also demonstrate that Bag5 interacts with the ER-resident chaperone GRP78/BiP and enhances its ATPase activity. Bag5 overexpression in 22Rv.1 prostate cancer cells inhibited ER-stress induced apoptosis in the unfolded protein response by suppressing PERK-eIF2-ATF4 activity while enhancing the IRE1-Xbp1 axis of this pathway. Cells expressing high levels of Bag5 showed reduced sensitivity to apoptosis induced by different agents while Bag5 downregulation resulted in increased stress-induced cell death.

**Conclusions:**

We have therefore shown that Bag5 is overexpressed in prostate cancer and plays a role in ER-stress induced apoptosis. Furthermore we have identified GRP78/BiP as a novel interaction partner of Bag5.

## Background

The Bag (Bcl-2 associated athanogene) protein family consists of 6 evolutionary conserved polypeptides (Bag1-Bag6) [1]. They share a common, C-terminal, single-copied BAG domain consisting of three alpha helices that interact with and modulate the activity of the molecular chaperone Hsp70 [2]. Structural biology and limited proteolysis studies identified the Bag domain as a 110-124 amino acid motif consisting of three antiparallel alpha helices of 30-40 amino acids each [2-4]. However the length of the Bag domain varies among the Bag family members, producing two distinct sub-groups: a ‘long’ Bag domain present in Bag-1 family of proteins and a ‘short’ Bag domain of Bag-3, Bag-4 and Bag-5 [5].

Several of the Bag proteins have been implicated in the control of apoptosis [6,7]. Bag-1 and Bag-3 (Bis) interact with Bcl-2 to reduce apoptosis induced by several factors [6,8]. Bag-4 (Sodd) associates with and blocks signaling of receptors of the tumor necrosis factor family [9,10]. Bag-6 (Scythe) modulates the nuclear pathway that communicates with mitochondria and regulates the release of cytochrome c thereby controlling apoptosis [11,12].

Other than the common Bag domain, Bag proteins do not share any homology in terms of sequence and encode for distinct domains: Bag-3 contains at its N-terminal region a WW domain [13] and a PXXP domain [14] while a ubiquitin-like domain is present in the central part of the Bag-1 proteins and in a double copy at the N-terminal part of Bag-6 [15]. Bag5 is exceptional in this group of polypeptides since it consists solely of 5 BAG domains structured in two and a half helices [5].

Bag proteins enhance cell proliferation and survival [16,17] and increase stress tolerance and therefore contribute to cancer development [18-20]. However the only function of Bag5 known so far is the inhibition of the activity of Hsp70 and the E3 ubiquitin ligase Parkin [21,22] in Parkinson’s disease. A possibility exists that like the other Bag proteins it may also be involved in tumor progression although this has not been demonstrated.

In this communication we show that Bag5 is overexpressed in prostate cancer and exerts an anti-apoptotic function. We further demonstrate that Bag5 is a stress inducible gene that functions as a co-chaperone of GRP78/BiP and that its increased expression results in increased resistance to UPR-induced apoptosis.

## Methods

### Antibodies

Rabbit polyclonal anti-eIF2α FL315, goat monoclonal anti-GRP78 (N20), mouse monoclonal anti-Bag5 (18Z) and anti-tubulin (TU-02) antibodies were purchased from Santa Cruz Biotechnology (Heidelberg, Germany). Rabbit polyclonal anti-GRP78 (ab21685) and anti-Bag5 (ab97660) antibodies were purchased from Abcam (Cambridge, UK). Rabbit polyclonal anti-GRP78 (ET21) and anti-β-actin antibodies were purchased from Sigma (Steinheim, Germany). Mouse antibody against HA (HA.11 clone 16B12) was purchased from Covance (Munich, Germany). Antibodies against PARP, ATF4, IRE1α, CHOP, phospho-IRE1α, phospho-eIF2α and PERK were purchased from Cell Signaling Technology (Frankfurt am Main, Germany). Anti-ATF6 antibody was purchased from Imgenex (Hamburg, Germany).

### Cell culture

All cell lines used in this work were purchased from ATCC.

22Rv.1, LNCaP and PC3 cells were cultivated in RPMI 1640 medium supplemented with 10% fetal calf serum (FCS). HEK293 cells were cultivated in Dulbecco´s modified Eagle´s medium (DMEM) supplemented with 10% FCS. RWPE-1, WPE-NB14 and WPE-NB26 cells were cultivated in keratinocyte serum free medium. All the cell culture media were purchased from Invitrogen (Karlsruhe, Germany).

### Cell treatments

Unless otherwise stated, cells were treated for the indicated time points with a final concentration of 300 nM thapsigargin (Life Technology, Hamburg, Germany), 10 μg/ml tunicamycin (Sigma, Steinheim, Germany), 20 μM fenretinide (Enzo Life Sciences, Lörrach, Germany), 10 μM (-)-epigallocatechingallate (EGCG, Santa Cruz, Heidelberg, Germany) and 10 nM Taxol (Sigma, Steinheim, Germany). Glucose starvation was performed cultivating the cells in glucose-free medium supplemented with 10% dialyzed FCS. Serum starvation was performed cultivating the cells in serum-free medium.

### Transfection experiments and siRNA

22Rv.1 and PC3 stably transfected with empty vector or Bag5 with FuGene6® (Promega, Mannheim, Germany). For the generation of stable pooled clones, cells were cultivated in RPMI supplemented with 10% FCS and 0.8 mg/ml G418 final concentration. HEK293 cells were transiently transfected with PromoFectin® (PromoKine, Heidelberg, Germany) according to the manufacturer’s recommendations. For siRNA experiment, cells were transfected with HiperFect (Qiagen, Hilden, Germany) with RNA antisense targeted against Bag5 or GFP as control. siRNA was purchased from Life Technologies (Darmstadt, Germany).

### Immunofluorescence

Immunofluorescence experiments were carried out on cells seeded in a 2-well glass slide (Lab-Tek® Chamber Slide System). After treatment with vehicle (ethanol 80%) or 300 nM thapsigargin, the medium was removed and the cells were stained with anti-Bag5 antibody and anti-PDI antibody (to track the ER). Samples were analyzed with a Leica TCS SPE confocal microscope. An IMARIS Coloc® (Bitplane, Zurich, Switzerland) module was used to calculate the co-localized voxels (volume unit, analogous to a pixel in two dimension images) between the two channels.

### Quantification of protein extracts

Protein concentration was quantified with the Bio-Rad Protein Assay (Bio-Rad, Munich, Germany) according to manufacturer’s instructions.

### Protein extraction

For protein extraction, cells were washed once with PBS 1X and resuspended in lysis buffer (50 mM Tris pH 8.0, 150 mM NaCl, 5 mM EDTA, 1% NP-40, 0.1% SDS, 1 mM PMSF). For protein extraction from patient material, 8 μm-thick frozen tissue sections were homogenized in lysis buffer with the TissueLyser (Qiagen, Hilden, Germany) and frozen at -80°C. Samples were centrifuged at 12000 × g for 10 min at 4°C, quantified, resuspended in sample buffer and boiled at 95°C for 5 minutes.

### Endoplamsic reticulum fractionation

Endoplasmic Reticulum fractionation was performed with the ER enrichment kit from Imgenex (Hamburg, Germany). After thapsigargin treatment cells were washed with PBS 1X by centrifugation at 2000 × g for 5 min. For homogenization, the cell pellet was resuspended in 1.5 ml of 1X isosmotic homogenization buffer supplemented with protease inhibitor cocktail and transferred into a glass tubes for the homogenizer (Braun, Melsungen, Germany). The samples were homogenized with the TissueLyser (Qiagen, Hilden, Germany). The homogenate was transferred into a new tube and centrifuged for 10 minutes at 1000 × g at 4°C to eliminate nuclei and cell debris. The supernatant was transferred into a new tube and centrifuged for 15 minutes at 12000 × g at 4°C to eliminate the mitochondria and the cell debris. The resulting supernatant was ultracentrifuged 1 h at 90000 × g for 1 h at 4°C. The pellet containing the ER was resuspended in 1X suspension buffer supplemented with protease inhibitor cocktail and dissolved by pipetting and vigorous vortexing.

### mRNA extraction and real time PCR

Total RNA was extracted with PeqGold (PeqLab, Erlangen, Germany) and first-strand cDNA synthesis was performed using the M-MLV reverse transcriptase (Promega, Mannehim, Germany) and random primers (Fermentas, St Leon-Rot, Germany). For q-RT-PCR analysis the Maxima SYBR Green/Rox qPCR Master Mix (Fermentas, St-Leon-Rot, Germany) and StepOne Plus Real-Time System apparatus (Applied Biosystems, Darmstadt, Germany) were used.

For Real Time PCR analysis the following primers were used: Bcl2, forward 5^′^-ATGTGTGTGGAGAGCGTCAACC-3^′^, reverse 5^′^-TGAGCAGAGTCTTCAGAGACAGCC-3^′^; Bax, forward 5^′^- CCTTTTCTACTTTGCCAGCAAAC-3^′^, reverse 5^′^- GAGGCCGTCCCAACCAC-3^′^; CHOP, forward 5^′^-TGGTCATTCCCCAGCCCGGG-3^′^, reverse 5^′^-TTCCCTGGTCAGGCGCTCGA-3^′^; Xbp1 spliced (Xbp1s) forward 5^′^-CCGGTCTGCTGAGTCCGCAGC-3^′^, reverse 5^′^-TGGCAGGCTCTGGGGAAGGG-3^′^; Bag5 forward 5^′^-TGTCCCCGGGTTTAGGGGTGTTC-3^′^, reverse 5^′^-TTCACAAGCACTGTCCCGCCC-3^′^; GRP78/BiP forward 5^′^-CGACCTGGGGACCACCTACT-3^′^, reverse 5^′^-TTGGAGGTGAGCTGGTTCTT-3^′^. Gene expression data analysis was normalized against the Ribosomal Protein 36 (Rib36). For Rib36 the forward 5^′^-GAAGGCTGTGGTGCTGATGG-3^′^ and reverse 5^′^-CCGGATATGAGGCAGCAGTT-3^′^ primers were used.

For Bag5 gene expression level in patient material, a set of 42 samples was used, including 15 benign prostatic hyperplasia (BPH) and 27 prostate cancer samples obtained from radical prostatectomy. The set of samples was obtained from the Tampere University Hospital (Tampere, Finland). The specimens were confirmed to contain >70% of malignant or non-malignant epithelial cells using hematoxylin and eosin-stained slides. Total RNA was extracted from the frozen sections with Trizol (Invitrogen, Hämeenlinna, Finland), and first-strand cDNA synthesis was performed using SuperScript III reverse transcriptase (Invitrogen, Hämeenlinna, Finland) and random primers (Fermentas, Glen Burnie, MA).

Bag5 gene expression was analyzed with the following primers: forward 5^′^-AGGTGTCCCCGGGTTTAG-3^′^ and reverse 5^′^-GATGTTGGTTTCCCATATCCA-3^′^. Values were normalized to β-actin using the primers: forward 5^′^-TGGGACGACATGGAGAAAAT-3^′^ and reverse 5^′^-AGAGGCGTACAGGGATAGCA-3^′^. For q-RT-PCR analysis the Maxima SYBR Green/Rox qPCR Master Mix (Fermentas, Helsinki, Finland) and CFX96 Real-Time System apparatus (Bio-Rad, Helsinki, Finland) were used.

### Statistical analysis

Unless otherwise stated, calculations of statistical significance in this work were performed according to Student’s t test. For comparison of the mean values in Bag5 gene expression study in BPH and Prostate Cancer patients one-way ANOVA and Bonferroni’s Multiple Comparison test were used. For comparison of the mean values of Bag5 stained cells in the tissue array analysis the Mann-Whitney test was used.

### Protein extraction from prostate tissue and tissue array

Radical prostatectomy specimens were obtained from patients undergoing surgery after prostate cancer diagnosis in the Tyrolean PSA Screening project for early detection of prostate cancer [23,24] and were worked up according to the standard histopathology protocol. The use of archive tumor tissue samples was approved by the Ethics Committee of the Innsbruck Medical University.

A tissue microarray containing tissue cores of 91 cancer cases was prepared as described in [25] and double stained with a polyclonal rabbit Bag5 antiboby (Imgenex, Hamburg, Germany) diluted 1:500 in Ventana diluent and a monoclonal antibody directed against the basal cell marker p63 (Clone 4A4 + Y4A3, Neomarkers, MS Cat 1084-P0) diluted 1:100 in Ventana diluent using a Ventany Discovery-XT staining automate (Ventana, Roche, Mannheim, Germany). Immunoreactivity was scored by an uropathologist (G.S.) considering the number of positive cells and the intensity of immunostaining for Bag5. Each case included 3 cores of tumor and 1 core of benign tissue. According to the percentage of positive cells a score from 0 to 4 was assigned to the case (0 = no staining; 1 = 10-25%; 2 = 25-50%; 3 = 50-75%; 4 = 75-100%). For each case, the value assigned to the tumor is the average of the three tumor cores. Immunostaining for the basal cell marker p63 present only in benign tissue served as a control for accurately distinguishing benign and tumor tissue.

### Colorimetric assay of ATP hydrolysis

ATP hydrolysis was measured using an ATPase assay kit from Innova Biosciences (Cambridge, UK). Briefly, 0.5 μg of purified GRP78 (StressMarq Biosciences, Victoria, Canada) was incubated in a buffer consisting of 0.5 M Tris pH 7.5 and 1 mM ATP in presence or absence of 0.17 μg of GST-Bag5 or GST-BagΔ 5 at 37°C. The experiment performed in presence only of GST-Bag5 was set as background control. After 30, 45 and 60 min, 50 μl PiColorLock™ Gold reagent and Accelerator were added to the solution. 2 minutes later 20 μl of stabilizer were added and the resulting green color was allowed to develop for 30 minutes at room temperature. Absorbance was measured at 595 nm. Enzymatic activity was calculated according to manufacturer’s instructions.

### Co-immunoprecipitation

For *in vivo* protein-protein interaction studies, co-immunoprecipitation experiment was performed by continuous rotation of protein A sepharose beads in TE buffer (10 mM Tris pH8 and 0,1 mM EDTA pH 8) with 4 μg of anti-GRP78/BiP specific antibody for 2 h at 4°C. HEK293 cells were treated with 2 nM Dithiobis(succinimidyl propionate) (DSP) in 10 ml PBS 1X for 30 minutes at room temperature (RT) to crosslink endogenous proteins. Crosslinking was stopped by the addition of 20 mM Tris pH7.5 for 15 minutes at RT. Thereafter cells were centrifuged at 2000 rpm for 5 minutes and the pellet lysed in 1 ml lysis buffer (50 mM Tris pH7,4, 120 mM NaCl, 1 mM EDTA, 0.4% NP-40). Cell lysate was sheered by passage 10 times through a 23 G needle (Braun, Melsungen, Germany), sonified (Amp 60, 10 pulses) and centrifuged for 10 minutes at 12000 × g at 4°C. The cell lysate and the beads were then incubated over night at 4°C boiled at 95°C and finally subjected to SDS-PAGE and western blot analysis.

### Caspase 3 cleavage measurement

Caspase 3 cleavage was measured with the CaspACE™ Assay System (Promega, Mannheim, Germany). 1·10^4^ cells were seeded in a 96 well plate and treated for 24 hours as indicated in the figure legend. At the end of the treatment, cells were lysed and Caspase 3 activity was measured according to manufacturer’s instructions.

## Results

### Bag5 is overexpressed in prostate cancer

**Figure 1:**
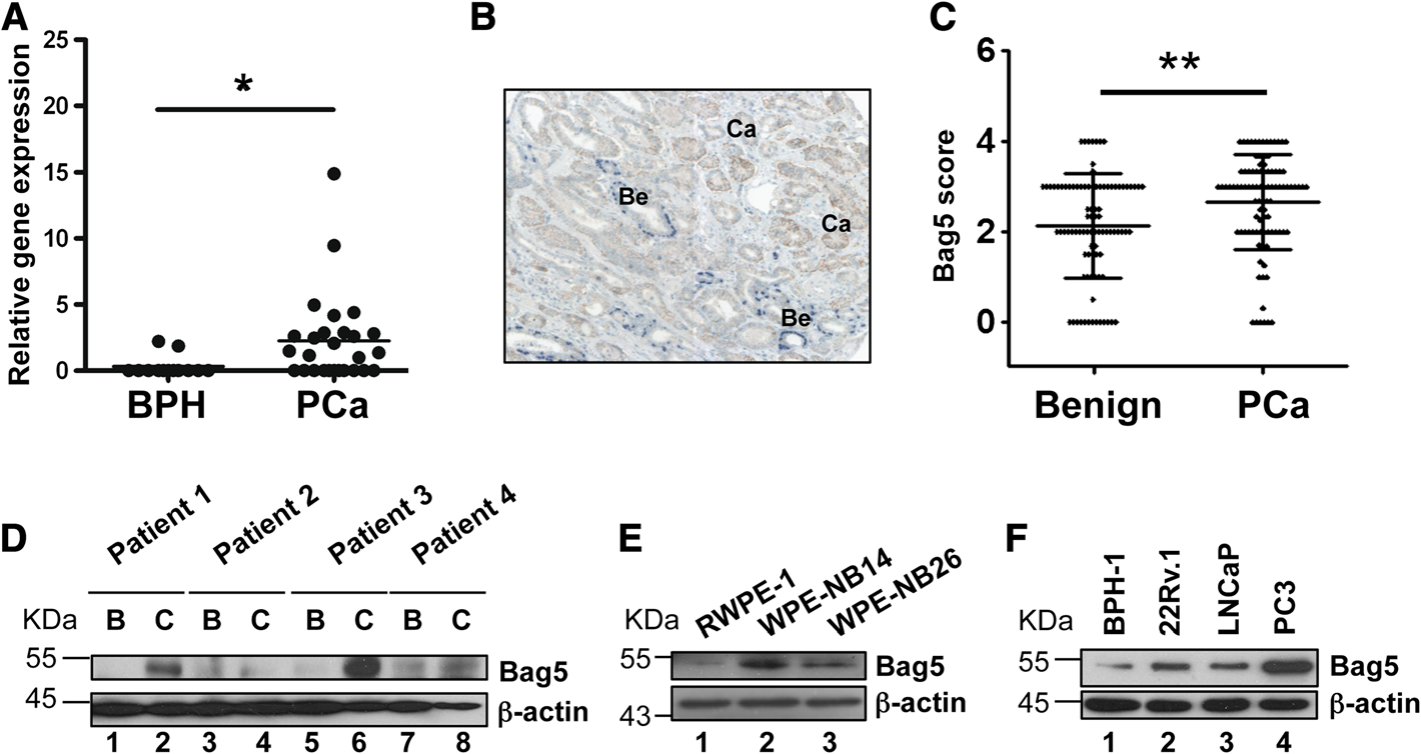
**Bag5 is overexpressed in prostate cancer.** Bag5 expression analysis in prostate cancer tissue and cell lines. **A**. Quantitative RT-PCR studies showing the relative expression of Bag5 in benign prostate hyperplasia (BPH, n = 15) and prostate cancer (PCa, n = 27). The expression values were normalized against the expression of β-actin. The horizontal line indicates arithmetic mean value. Non-parametric Kruskal-Wallis with Dunn’ post-test was used to analyze the statistical significance of the differences between the groups (* = p <0.05). **B**. Representative immunohistochemical staining used in the tissue array analysis. P63 (blue) and Bag5 (brown) staining are shown. Bening (Be) and cancer (Ca) tissue is indicated. **C**. Tissue microarray containing tissue cores of 91 cancer cases. The horizontal line indicates arithmetic mean value. (**p < 0.001). **D**. Western blot analysis of Bag5 protein expression in prostate cancer patients comparing cancer (C) and surrounding benign tissue (B). β-actin was used as equal loading control. **E**. Western blot analysis of Bag5 protein expression in an *in vitro* prostate tumor progression model using RWPE-1 (benign), WPE-NB14 (primary tumor) and WPE-NB26 (metastatic tumor) cells. β-actin was used as equal loading control. F. Western blot analysis of Bag5 protein expression in prostate cell lines. Equal loading control was checked with β-actin.

To determine whether Bag5 plays a role in prostate cancer development, we first analyzed its expression in benign prostatic hyperplasia (BPH) and compared it with its expression in prostate cancer. We observed at the RNA level that only 13% of the BPH samples (2/15) expressed Bag5 compared to 59% tumor probes (16/27) (Figure 1A). In addition immunohistochemical analysis of Bag5 was performed on a tissue microarray containing benign and malignant prostate tissues from a core of 91 cancer cases (Figure 1B). Staining score of the percentage of cells positive for Bag5 was significantly increased in the malignant compared to the benign tissues (Figure 1C). Furthermore, analysis of Bag5 expression was performed in an immunoblot assay on Gleason 9 prostate cancers and their corresponding benign surrounding area. In this study, no Bag5 expression was detected in the benign biopsies while 3 out of the 4 cancer samples analyzed (75%) were positive for Bag5 (Figure 1D). These results demonstrate that Bag5 expression is increased in prostate cancer both at the RNA and protein level.

The tumor-specific expression of Bag5 was not restricted to biopsies but could be reproduced in a cell culture model of prostate cancer progression where RWPE-1, WPE-NB14 and WPE-NB26 represent different stages of malignancy from benign to a more aggressive prostate tumor state (reviewed in [26]). Here again, increased Bag5 expression was found in the tumor compared to the benign cell lines (Figure 1E). In addition we could show in established prostate cell lines that Bag5 expression is high in the more aggressive PC3 cells compared to less aggressive 22Rv.1 and LNCaP cells and the benign-prostatic hyperplasia (BPH-1) derived cells (Figure 1F).

### ER stress enhances Bag5 expression and alters its cytoplasmic localization

**Figure 2:**
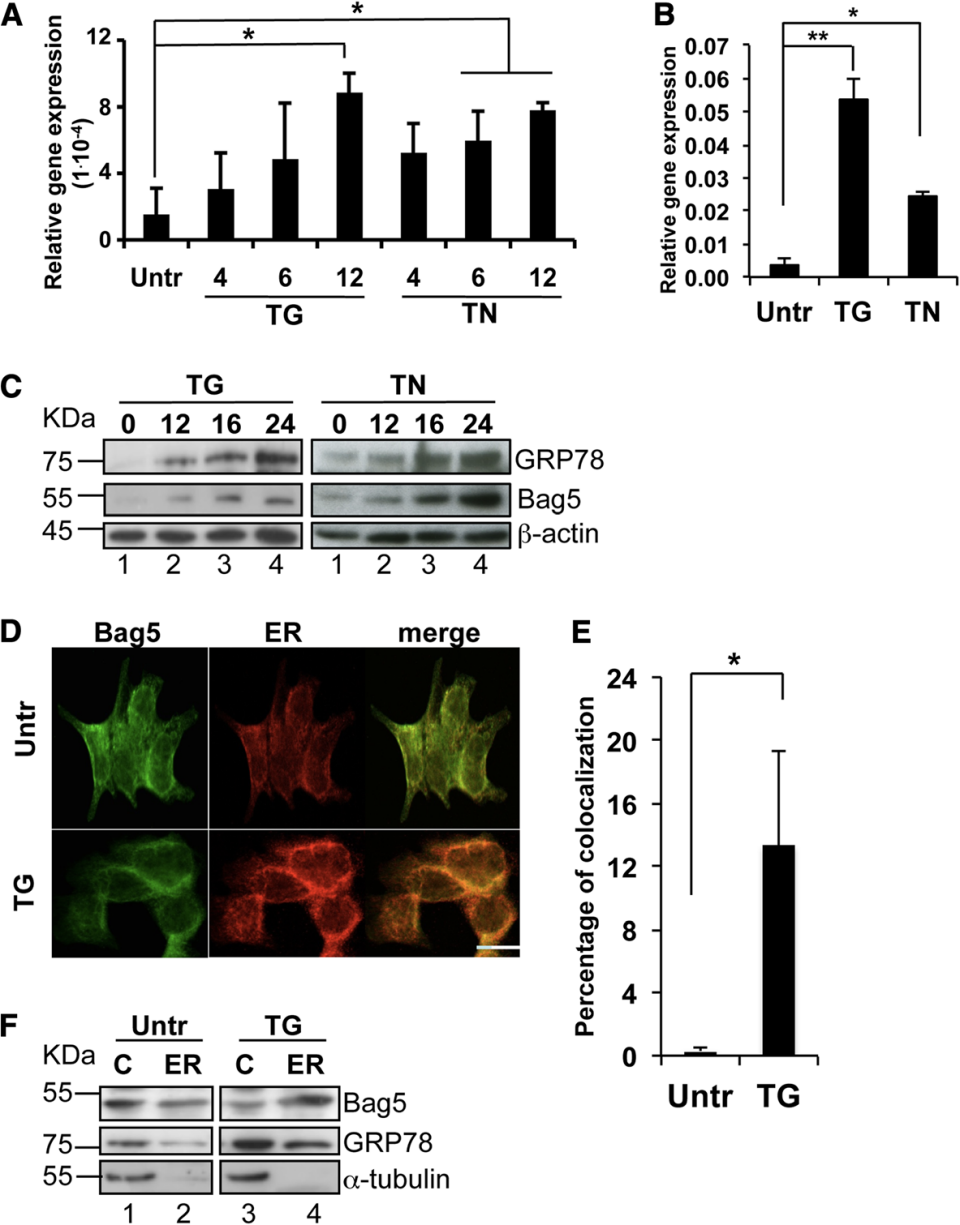
**Bag5 is stress-induced and accumulates in the endoplasmic reticulum.** Bag5 gene expression is induced upon stress. **A**. Real time PCR analysis of Bag5 gene in 22Rv.1 cells upon treatment with thapsigargin (TG) or tunicamycin (TN) for the indicated time points. Gene expression values were normalized to Rib36. Bar charts indicate the mean value of three independent experiments ± SD. (* p < 0.05) **B**. Real time PCR analysis of GRP78 gene in 22Rv.1 cells upon treatment with thapsigargin (TG) or tunicamycin (TN) for 12 h. Gene expression values were normalized to Rib36. Bar charts indicate the mean value of three independent experiments ± SD. (* p < 0.05; ** p < 0.01). **C**. Bag5 protein expression is induced upon stress. Western blot analysis of 22Rv.1 cell extracts after thapsigargin or tunicamycin treatment for the indicated time points. β-actin was detected as loading control. D. Bag5 accumulates into the ER upon stress. **D**. Bag5 associates to the ER upon stress. Confocal microscopic analysis of 22Rv.1 cells-paraformaldehyde-fixed and stained with an anti-Bag5 antibody (green channel) or the ER-tracker (red channel) after 12 hours thapsigargin treatment. All images (40X) were acquired with a Leica TCS SPE confocal microscope (Leica Microsystems). The scale bar indicates 25 μm. **E**. Quantification of the percentage of co-localization performed with the software Imaris CoLoc. Bar charts indicate the average of three fields of three independent experiments ± SD (* p < 0.01). **F**. ER extraction from 22Rv.1 cell lysates treated with TG for 8 h. Western blot analysis was performed with a Bag5 or a GRP78 specific antibody. Anti-α-tubulin was used for ER fractions quality control (C: cytoplasm; ER: endoplasmic reticulum).

Previous reports have implicated the Bag proteins in the development of stress tolerance [19,27], one of the key features for cancer growth and chemoresistance. We therefore investigated whether Bag5 expression levels are influenced by stress. Treatment of 22Rv.1 cells with the stress inducers thapsigargin (TG) or tunicamycin (TN) up to 12 h resulted in a significant increase in Bag5 mRNA expression (Figure 2A). The increased expression of Bag5 following TG and TN treatment occurred concomitantly with an increased expression of the ER-chaperone GRP78/BiP (Figures 2B). Bag5 and GRP78/BiP protein levels were also increased following treatment of the 22Rv.1 cells with TG and TN (Figure 2C). Similar results were obtained in the metastatic cell line PC3 that as previously shown in Figure 1F expresses high levels of Bag5 (Additional file 1: Figure S1).

Stress induction did not only increase Bag5 expression, but it also modified its subcellular localization. In resting conditions Bag5 showed a diffuse staining in the cytoplasm. However when cells were treated for 12 hours with TG, Bag5 staining became perinuclear and a strong co-localization with the ER was observed as determined by the use of the ER tracker (Figure 2D). Quantification of the co-localization in three fields of three independent experiments making use of the software IMARIS Coloc® showed that indeed Bag5 was significantly enriched in the ER after stress induction (Figure 2E). This result was confirmed by a fractionation experiment in which it could be shown that already after 8 h of TG treatment the ratio of Bag5 in the ER compared to the cytoplasm was substantially increased (Figure 2F). A similar increase in the distribution of GRP78/BiP in the ER was also observed (Figure 2F).

### Bag5 interacts with GRP78/BiP

**Figure 3:**
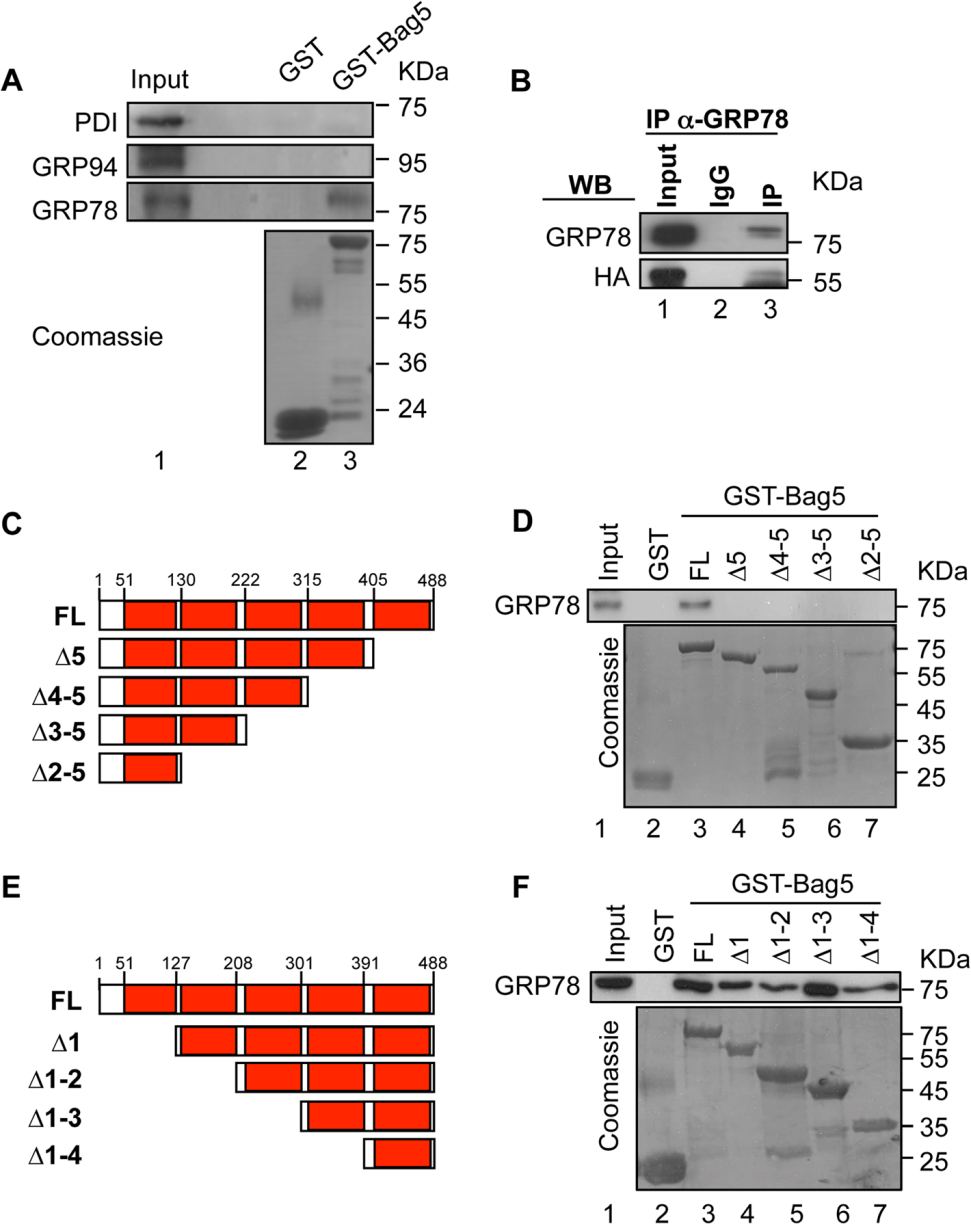
**Bag5 interacts with GRP78/BiP. A**. Bag5 interacts with GRP78/BiP but not with GRP94 and PDI. GST-pull down assay performed incubating 10 μg of GST-Bag5 and 250 μg of 22Rv.1 cell lysate. Equal recombinant protein employed in the assay was checked by staining the membrane with coomassie. 5% of the lysate was loaded as input. **B**. Bag5 and GRP78 interact *in vivo*. Co-immunoprecipitation assay performed in HEK293 cells transfected with HA-Bag5. GRP78/BiP was immunoprecipitated with an anti-GRP78 specific antibody and IgG was used as negative control. Western blot analysis was performed with an anti-HA and anti-GRP78 antibody. **C-F**. The fifth Bag domain of Bag5 mediates the interaction with GRP78/BiP. **C** and **E**. Diagrammatic representation of Bag5 and the deletion mutants used for the GST-pull down assay. **D** and **F**. GST-pull down assay performed incubating 10 μg of GST-fused protein and 500 μg of 22Rv.1 cell lysate. An anti-GRP78 antibody was used for western blot analysis. The membrane was stained with coomassie for equal protein amount employed in the experiment.

Since Bag5 localizes at least partially in the endoplasmic reticulum, we wanted to investigate whether as co-chaperone was able to interact with the major chaperones in this organelle. GST-pull down assay was therefore carried out with GST-fused Bag5 and lysates of 22Rv.1 prostate cancer cells. Western blot analysis with specific antibodies showed that Bag5 interacted with GRP78/BiP but it did not bind other ER chaperones such as protein disulfide isomerase (PDI) or GRP94 (Figure 3A). The interaction of Bag5 and GRP78/BiP was confirmed in a co-immunoprecipitation assay in HEK293 cell transfected with HA-Bag5 (Figure 3B).

As Bag5 is made up of five BAG domains, it was necessary to determine whether all the five domains bind equally to GRP78/BiP. A GST-pull down assay was therefore performed with 22Rv.1 cell lysate and GST-fused carboxy- and amino-terminal deletion mutants of Bag5 that sequentially deleted one BAG domain at a time (Figure 3C and E). Deletion of the fifth BAG-domain abrogated binding of GRP78/BiP to the full length Bag5 protein while sequential deletion of the N-terminal sequences up to the fifth BAG domain modulated but did not abolish binding to GRP78/BiP (Figures 3C and D). This confirmed that the fifth BAG domain is responsible for binding to GRP78/BiP.

### Bag5 modulates GRP78/BiP activity

**Figure 4:**
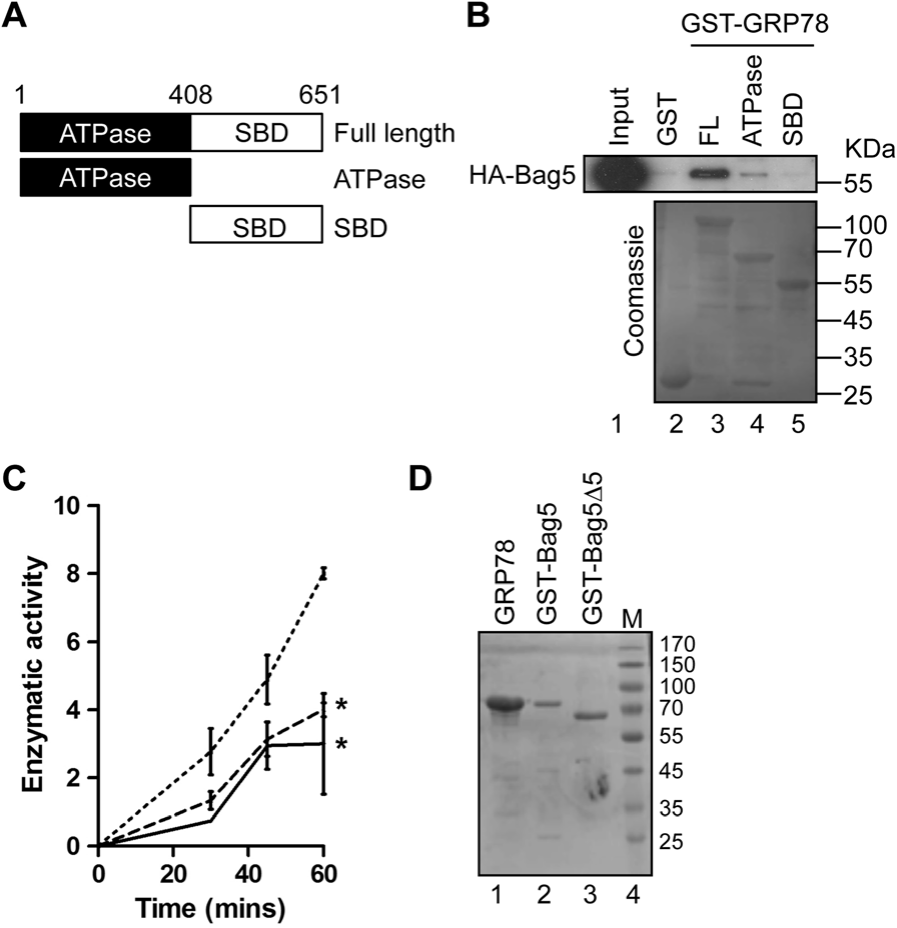
**Bag5 functions as co-chaperone of GRP78/BiP. A**. Diagrammatic representation of the molecular chaperone GRP78/BiP and its domains. Numbers indicate the amino acid position. **B**. Bag5 binds the ATPase domain of GRP78. GST-pull down assay was performed incubating 10 μg of GST-fused protein with 500 μg of HEK-293 cells transfeced with HA-Bag5. A specific anti-HA antibody was used in western blot analysis to detect the binding. Equal recombinant protein employed in the assay was checked by coomassie staining. **C**. Bag5 enhances GRP78/BiP ATP hydrolysis. ATPase assay with purified GRP78 in absence or presence of Bag5 (dashed line) or Bag5∆ 5 (dotted line). Results are expressed as the average of three independent experiments ± SD (* p < 0.05) **D**. Coomassie staining of the proteins employed in the ATPase assay.

To determine the domains of GRP78/BiP to which Bag5 binds, GST-pull down studies were carried out with GST-ATPase and GST-substrate binding domains of GRP78/BiP along with extracts from HEK293 cells previously transfected with an HA-Bag5 construct. These studies show that Bag5 interact with the ATPase domain of GRP78 but not the SBD (Figure 4B). Binding of Bag5 to the ATPase binding domain of GRP78/BiP suggests that Bag5 may affect the ATPase activity of GRP78/BiP. In an *in vitro* ATPase hydrolysis assay we could show that the addition of Bag5 enhanced the ATPase activity of GRP78/BiP but not Bag5 mutant lacking the fifth Bag domain (Bag5Δ 5) (Figure 4C) although both proteins employed in the assay were expressed at equal levels (Figure 4D).

### Bag5 expression levels modulate the unfolded protein response

Stressful conditions such as nutrient starvation, hypoxia or changes in pH to protect cells and promote cell survival activate a signaling pathway known as the UPR [28]. However, when stressful conditions are prolonged, the UPR induces apoptosis by shutting down protein synthesis [29]. Since the UPR is regulated by GRP78/BiP [30] and Bag5 binds this protein, we investigated if alteration of the level of Bag5 would affect the UPR. We overexpressed Bag5 by stable transfection in 22Rv.1 cell with an HA-Bag5 or an empty vector as control and induced the UPR by exposure of these cells to thapsigargin for 6 and 12 h.

**Figure 5:**
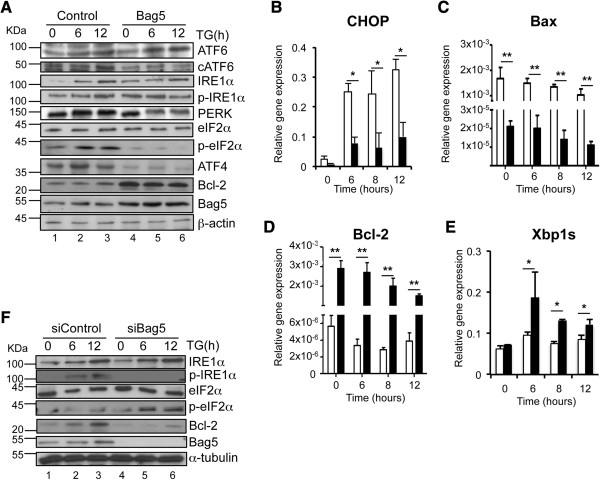
**Bag5 overexpression modulates the unfolded protein response. A**. 22Rv.1 stably overexpressing Bag5 showed increased IRE1/Xbp1 axis activation. Western blot analysis of 22Rv.1 cells stably transfected with pcDNA3.1-HA-Bag5 or the empty vector as control. After treatment with TG for the indicated time points, cells were lysed and subjected to western blot analysis using the indicated antibody. As equal loading control an anti-β-actin antibody was used. **B** - **E**. Real time-PCR analysis of 22Rv.1 cells stably expressing the empty vector control (open bars) or HA-Bag5 (filled bars) treated with TG for the indicated time points. Gene expression analysis was performed for CHOP (B), Bcl-2 (C), Bax (D) and the spliced form of Xbp1, Xbp1s (E). Values were normalized to Rib36. Bar charts indicate the mean of at least three independent experiments ± SD (*p <0.05; ** p < 0.01). **F**. Western blot analysis of 22Rv.1 cells transfected with siRNA targeting GFP or Bag5 and treated with TG for the indicated time points. Anti-α-tubulin was used as equal loading control.

GRP78/BiP [30] regulates the UPR by activating the kinase/endonuclease IRE1α, the transcription factor ATF6 and the kinase PERK. While the first two branches promote cell survival, the third is responsible for apoptosis induction [31,32]. Overexpression of Bag5 resulted in decreased cleavage of the transcription factor ATF6 (cATF6) while it produced an increase in the basal levels of IRE1α phosphorylation (Figure 5A). In addition Bag5 overexpression reduced the expression of the kinase PERK resulting in a reduced phosphorylation of the PERK downstream target eIF2α and of the transcription factor ATF4 (Figure 5A). These observations correlated with a Bag5-mediated decrease in expression of the transcription factor CHOP/GADD153 since it is regulated by ATF4 (Figure 5B). In addition we could also observe increased expression of the anti-apoptotic factor Bcl-2, a gene suppressed by CHOP (Figure 5A). As CHOP triggers apoptosis by inducing Bax and suppressing Bcl-2 gene expression [33], we also analyzed the expression of these two genes. Bag5 overexpression resulted not only in a decreased CHOP/GADD153 (Figure 5B) but also in a decreased Bax and an increased Bcl-2 expression (Figures 5C and D) gene expression. Furthermore since an increase in the phosphorylation of the kinase/endonuclease IRE1α was observed upon Bag5 overexpression, we analyzed the splicing of the target Xbp1 by RT-PCR (Xbp1s). As shown in Figure 5E, Xbp1 splicing was increased in Bag5 clones compared to the vector control.

To confirm these results, Bag5 expression was reduced in 22Rv.1 cells in a siRNA knock down experiment and the cells were treated for 6 and 12 h with thapsigargin (TG) to activate the UPR. Consistent with the overexpression results, a knock down of Bag5 expression is expected to produce the reverse results. Indeed the downregulation of Bag5 expression resulted in an increased eIF2α but a decreased IRE1α phosphorylation (Figure 5F). Taken together these results indicate that Bag5 induces the IRE1α pro-survival branch while it inhibits the PERK-eIF2α-ATF4 pro-apoptotic axis.

### Bag5 overexpression increases stress tolerance in prostate cancer cells

**Figure 6:**
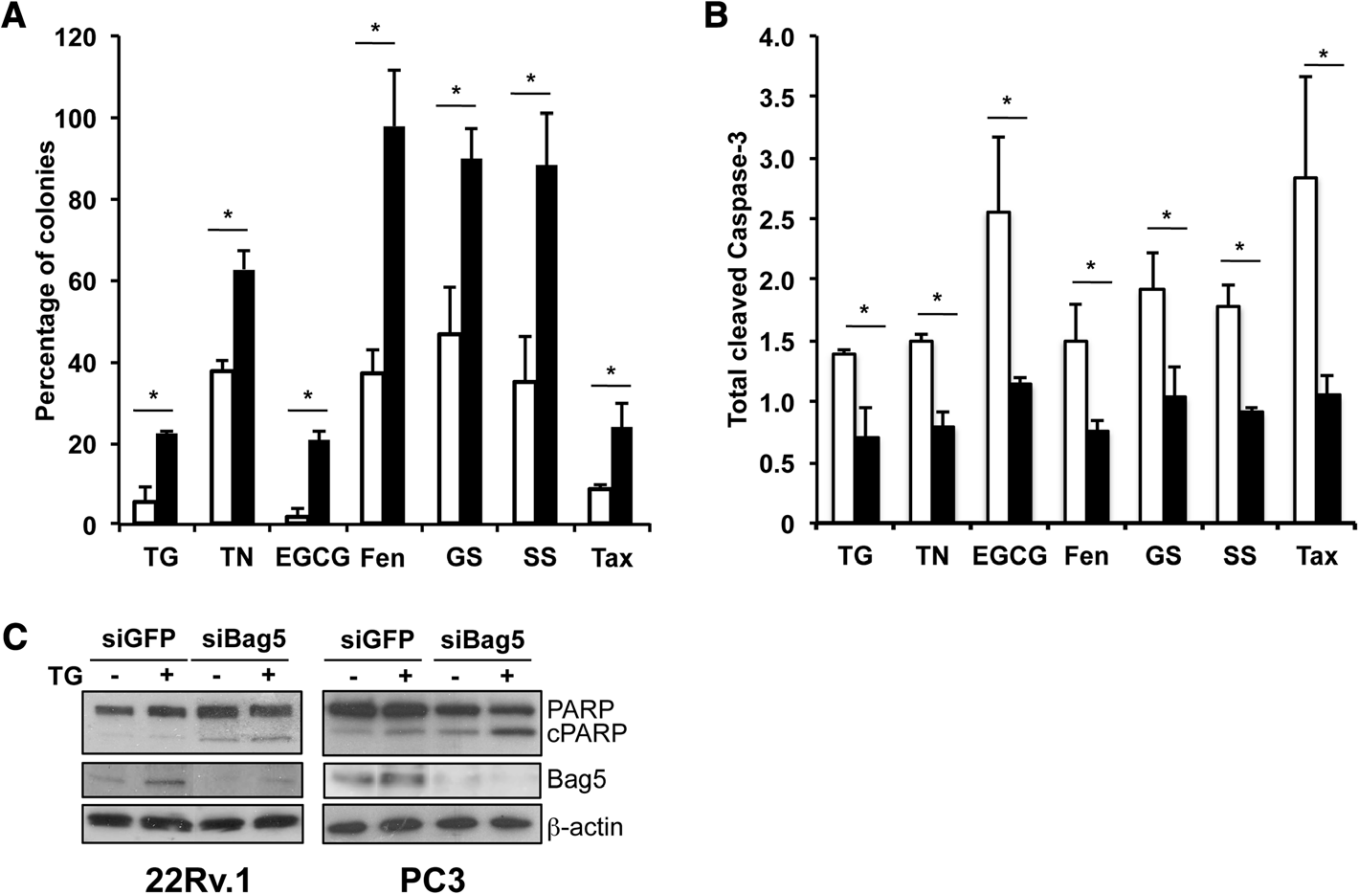
**Bag5 increases prostate cancer cell chemoresistance.** Modulation of Bag5 levels influences prostate cancer cell response to chemotherapy. **A** and **B**. Colony forming assay (A) and caspase-3 cleavage analysis (B) of 22Rv.1 cells stably expressing the empty vector control (open bars) or HA-Bag5 (filled bars). Bar charts represent the average of three independent experiments ± SD. * p < 0.05. (TG: thapsigargin, 75 nM; TN: tunicamycin, 10 μg/ml; EGCG: (-)-epigallocatechingallate, 10 μM; Fen: fenretinide, 20 μM; GS: glucose starvation; SS: serum starvation; Tax: taxol, 10 nM). * p < 0.05; ** p < 0.01. **C**. Western blot analysis of 22Rv.1 and PC3 cells transfected with siRNA targeting Bag5 or GFP as control and treated with 300 nM TG for 24 hours. Anti-β-actin antibody was used as equal loading control.

To determine if the anti-apoptotic activity of Bag5 is specific to thapsigargin, a clonogenic assay was performed with 22Rv.1 cells transfected with a Bag5 or an empty expression vector and treated with several stress-inducing compounds such as tunicamycin, (-)-epigallocatechingallate (EGCG), feneretinide, taxol or glucose- or serum-starved. Overexpression of Bag5 in all the cases analyzed increased survival of the cells compared to the vector control cells (Figure 6A). In addition we could show that stress-induced apoptosis assayed by caspase-3 cleavage in the same culture conditions was decreased in the Bag5 transfected cells compared to the control cells. In the case of thapsigargin treatment we could show the converse experiment in that siRNA knock down of Bag5 induced apoptosis measured by PARP cleavage (Figure 6C). This result was even more convincing for the metastatic cell line PC3 (Figure 6C), where Bag5 levels are higher compared to the 22Rv.1 (Figure 1F). These studies demonstrate that Bag5 protects against stress-induced apoptosis.

## Discussion

In this work we showed that Bag5 interacts with the molecular chaperone GRP78/BiP demonstrating that in addition to the function of Bag proteins as interaction partners of Hsp70/Hsc70, a member of this family (Bag5) additionally interacts with the ER-resident chaperone GRP78/BiP. This expands the network of interaction partners of the Bag family of co-chaperones. In addition we showed for the first time at the RNA and protein levels that Bag5 is overexpressed in prostate cancer and that it plays a role as a pro-survival factor in UPR-induced apoptosis.

### Bag5 is a co-chaperone of GRP78/BiP and promotes cancer cell survival

Bag proteins have been described to interact with the molecular chaperone Hsp/Hsc70 [2] but recently it is reported that Bag-1 interacts with the ER chaperone GRP78/BiP [34] suggesting that other Bag proteins may share this property. In this study we could show that Bag5 also interacts with GRP78/BiP confirming that the Bag proteins may be more versatile in their interactions with molecular chaperones. However Bag5 does not interact with all ER-resident chaperones, it does not interact with the protein disulfide isomerase PDI or the Hsp90 homolog GRP94 showing selectivity in its interaction partners. The observation that Bag5 interacts with GRP78 and stimulates its ATPase activity expands the range of action of the Bag proteins in other cellular events such as ER-mediated stress response and the UPR.

From the analysis of the effect of Bag5 in the UPR, we could show that it preferentially stimulates IRE1α while it suppresses PERK/eIF2α pathways resulting in growth advantage for the cells. The action of Bag5 in regulating events in the ER is consistent with its increased association to this cellular compartment following thapsigargin treatment even if the mechanism by which it is recruited to the ER is not clear and needs further investigation. Intriguingly we could also observe that ectopic expression of Bag5 resulted already in untreated cells in increased Bcl-2 and decreased Bax expression independent from stress induction. It is possible therefore that Bag5 could contribute to cell survival both by interacting with GRP78 enhancing its enzymatic activity and by modulating Bcl-2/Bax ratio and that these two events could be independent from each other.

### Bag5: a new tumor marker?

RNA and protein expression studies presented in this work show that the expression of Bag5 is increased in malignant compared to benign prostate tissue. Since other Bag family members are overexpressed in several types of cancers in addition to prostate cancer [35-37], such as breast [38,39], colon [40] and pancreatic [41,42] cancers, it is likely that increased Bag5 expression would be found in other type of tumors as well. If this is the case, it would be worth investigating the use of Bag5 as a novel tumor biomarker.

From the results of this work that Bag5 is a stress-inducible gene and it is anti-apoptotic, we would expect a tumor with high expression of Bag5 to be more aggressive and less responsive to stress-inducing chemotherapeutic agents. This hypothesis is supported by our observation that up- or downregulation of Bag5 levels modifies the ability of prostate cancer cells to respond to stress. This agrees with finds that Bag5 gene expression is increased in rat livers upon exposure to epatotoxants [43] and that it is induced in MCF7 breast cancer cells [44] and ovarian cancer spheroids [45] upon taxol treatment.

## **Conclusions**

These finding together with the work described in this communication identify Bag5 as a gene whose expression is regulated by chemotherapeutic drugs and an anti-apoptotic gene. In addition we showed that Bag5 interacts with the molecular chaperone GRP78/BiP, often found overexpressed in chemoresistant tumors (reviewed in [46]). Downregulating Bag5 levels and/or interfering with Bag5-GRP78/BiP interaction could therefore represent a novel therapeutic approach to overcame chemoresistance and to treat late stage tumors.

## Competing interests

The authors declared that they have no competing interests.

## Authors’ contribution

Conceived and designed the experiments: DM, AB, CR, TV, HK. Performed the experiments: DM, AB, CR, TVW, GS, SL. Wrote the paper: DM and ACBC. All authors read and approved the final manuscript.

## Pre-publication history

The pre-publication history for this paper can be accessed here:

http://www.biomedcentral.com/1471-2407/13/96/prepub

## Supplementary Material

Additional file 1: Figure S1 Bag5 is stress-induced in PC3 cell lines. Click here for file
