# Supplementary material for: Bcl-2 associated athanogene 5 (Bag5) is overexpressed in prostate cancer and inhibits ER-stress induced apoptosis
Source: BMC Cancer. 2013 Mar 1;13:96. doi: 10.1186/1471-2407-13-96 (PMC3598994; doi:10.1186/1471-2407-13-96)
Supplement: Additional file 1: Figure S1 — Bag5 is stress-induced in PC3 cell lines. [file 1471-2407-13-96-S1.docx]

**
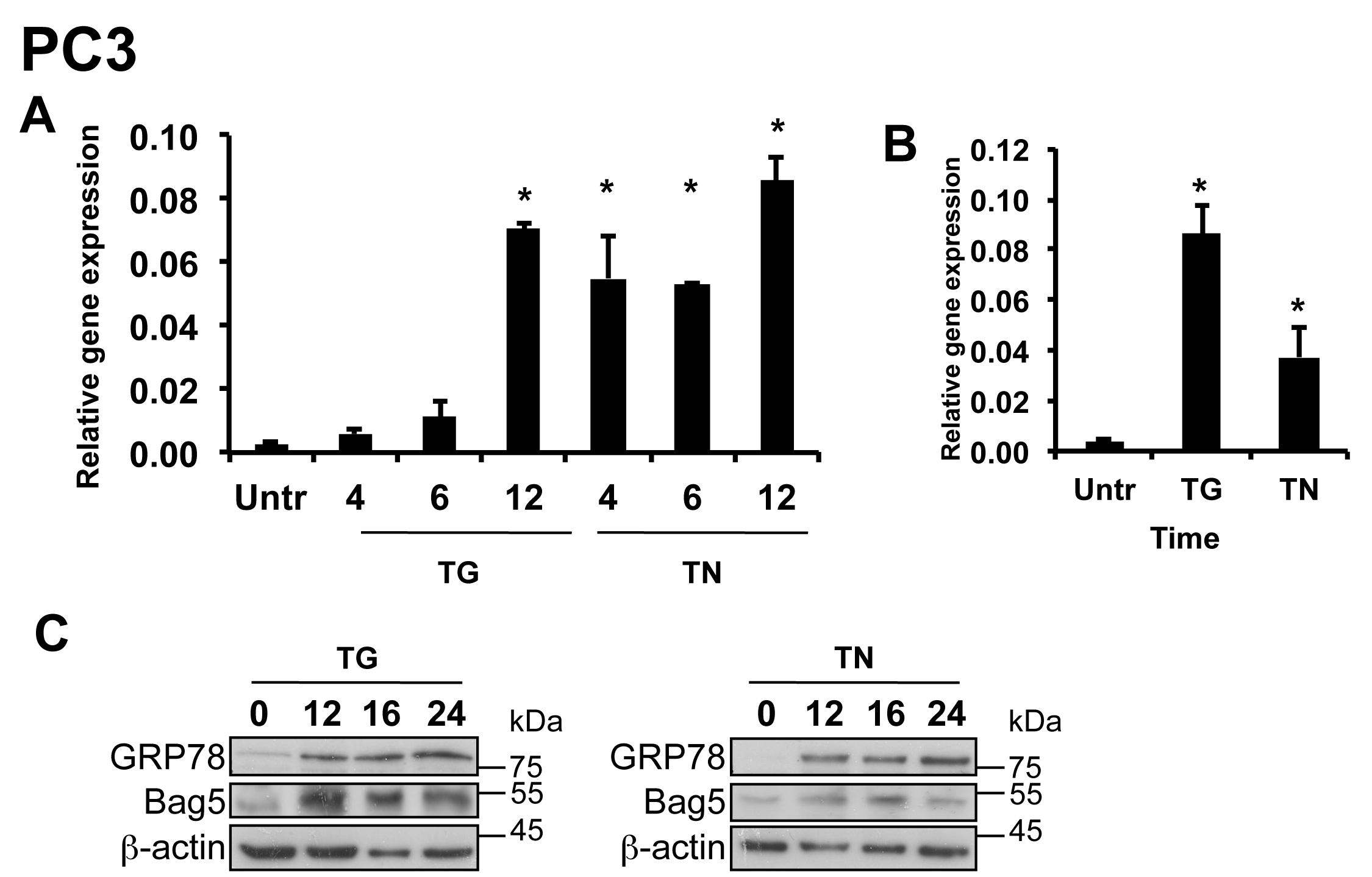
**

**Supplementary figure 1.**

**Bag5 is stress-induced in PC3 cell lines.**

Bag5 gene expression is induced upon stress in PC3 cells. A. Real time PCR analysis of Bag5 gene in PC3 cells upon treatment with 300 nM thapsigargin (TG) or 10 µg/ml tunicamycin (TN) for the indicated time points. Gene expression values were normalized to Rib36. Bar charts indicate the mean value of three independent experiments ± SD. (* p<0.05) B. Real time PCR analysis of GRP78 gene in PC3 cells upon treatment withthapsigargin (TG) or tunicamycin (TN) for 12h. Gene expression values were normalized to Rib36. Bar charts indicate the mean value of three independent experiments ± SD. (* p<0.05; ** p<0.01). C. Bag5 protein expression is induced upon stress. Western blot analysis of PC3 cell extracts after thapsigargin or tunicamycin treatment for the indicated time points. β-actin was detected as loading control.
